# Electrically tunable plasmonic metasurface as a matrix of nanoantennas

**DOI:** 10.1515/nanoph-2023-0796

**Published:** 2024-02-27

**Authors:** Luis Angel Mayoral Astorga, Masoud Shabaninezhad, Howard Northfield, Spyridon Ntais, Sabaa Rashid, Ewa Lisicka-Skrzek, Hamid Mehrvar, Eric Bernier, Dominic Goodwill, Lora Ramunno, Pierre Berini

**Affiliations:** School of Electrical Engineering and Computer Science, 6363 University of Ottawa, Ottawa, ON K1N 6N5, Canada; 6363 NEXQT Institute, University of Ottawa, 25 Templeton Street, Ottawa, Ontario K1N 6N5, Canada; School of Electrical Engineering and Computer Science, 6363 University of Ottawa, Ottawa, Canada; Canadian Centre for Electron Microscopy, McMaster University, Hamilton, Canada; Huawei Technologies Canada, Ottawa, Canada; Department of Physics, 6363 University of Ottawa, Ottawa, ON K1N 6N5, Canada

**Keywords:** optical, reflectarray, plasmonics, metasurface, indium tin oxide, phased array

## Abstract

We report the fabrication and characterization of a plasmonic metasurface comprising electrically-contacted sub-wavelength gold dipole nanoantennas, conformally coated by a thin hafnia film, an indium tin oxide layer and a backside mirror, forming metal–oxide–semiconductor (MOS) capacitors, for use as an electrically-tunable reflectarray or metasurface. By voltage biasing the nanoantennas through metallic connectors and leveraging the carrier refraction effect in the MOS capacitors, our measurements demonstrate phase control in reflection over a range of about 30°, with a constant magnitude of reflection coefficient of 0.5, and the absence of secondary lobes. Comprehensive electromagnetic and quantum carrier models of the structure are developed and are in excellent agreement with the measurements. The metasurface holds promise for use as an optical phased array.

## 1 Introduction

Electrically-tunable metasurfaces hold significant promise for numerous applications, including dynamic beam forming and focusing, beam steering, and, generally, wavefront shaping [1], [2], [3].

Beam steering, for example, is key to LIDAR scanners for self-driving cars and autonomous machines, but is typically achieved using mechanical means, leading to bulky and slow systems which precludes their use in cases that require a high refresh rate. Nanophotonic approaches hold promise for LIDAR applications [4], particularly to implement beam steering devices based on optical phased arrays [5], [6]. Compared to mechanical scanning, optical phased arrays exploiting electronic tuning can enable significantly faster scanning speeds.

An ideal beam steering metasurface should possess several key characteristics [6]. It should feature a large aperture, allowing for the steering of light across a wide range of angles, without generating diffraction orders. Simultaneously, it should maintain the beam at a constant magnitude with low loss throughout the steering process. In addition, such a surface should be reliable, compact, cost-effective, and solid-state, eliminating the need for mechanical components. It should also offer rapid scanning capabilities to enable swift operations. Optical phased arrays for beam scanning can be implemented using tunable metasurfaces [6]. Such structures typically consist of a planar arrangement of phase-tunable coherent light emitters, where an individual emitter is referred to as a pixel. A pixel can take various forms and be arranged to form a metasurface that operates in reflection (reflectarray) or in transmission (transmitarray).

Optical phased arrays based on plasmonic nanoantennas hold promise for meeting the requirements of optical beamsteering. Resonant plasmonic nanoantennas support strongly enhanced fields which confer a high sensitivity to local perturbations in refractive index. They are also nanoscale structures, enabling pixels to be dimensioned to sub-wavelength scales which preclude the emergence of grating (secondary) lobes. Moreover, metal nanoantennas can also serve as device electrodes, ensuring strong optical overlap with the active region.

Integrating plasmonic nanostructures with a semiconductor enables exploitation of the carrier refraction effect through electrical biasing in an appropriate device configuration. Altering the carrier density by applying a gate voltage provides a pathway to dynamically and actively tune the phase, amplitude, and polarization of the emerging light [6], [7], [8], [9], [10]. A promising approach to dynamically tune a metasurface for beam steering involves manipulating the optical properties of a semiconductor used in its construction. Transparent conductive oxides (TCOs), particularly indium tin oxide (ITO), are of strong interest as this material offers high transparency in the visible and near-infrared spectrum, coupled with excellent electrical conductivity, making it an ideal candidate for applications in photonics and optoelectronics.

Implementing devices that replicate the function of a turning mirror to control a beam generated by a nearby source involves the pixelation and application of a controllable phase gradient across a reflective area. Such a device, termed a tunable reflectarray, allows for steering a monochromatic beam to any desired angle of reflection. To achieve this, it is desirable that the phase of each pixel over the beam cross-section to be continuously variable from 0 to 2*π* without affecting the incident light’s intensity.

The versatility of the tunable reflectarray concept makes it a powerful tool. Much of the ongoing research in beam steering devices, particularly those involving optical metasurfaces or plasmonic nanostructures, is focused on the development of reflectarrays constructed with tunable pixels, incorporating ITO as electro-optical active material.

Huang et al. [11] achieved a phase change of 184° at a wavelength of 1573 nm for a single-gated device comprised of 50 nm thick gold strips on 5 nm of Al_2_O_3_, on 20 nm of ITO, on 80 nm of Au. However, their structure displayed a strong dependence of reflectance on the bias voltage. Park et al. [12] reported phase tuning of 180° in the reflected field using a device stack comprised of Au, Al_2_O_3_, ITO and Au, operating at a wavelength of 6 μm. Double-gated devices were reported where the metasurface elements were controlled with two independent voltages driving two MOS field effect regions, thereby enabling wider phase tunability [13]. They employed ITO as the active metasurface material and a composite hafnia/alumina gate dielectric. The device exhibited a continuous phase shift from 0 to 303° at a wavelength of *λ* = 1550 nm, however, it displayed a significant dependence of reflectance on the bias voltage.

More recently, Kim et al. [14] introduced a beam steering device composed of a 5 nm thick ITO layer sandwiched between two insulator layers (Al_2_O_3_/HfO_2_). By employing two separate bias controls, they managed to control both the amplitude and the phase from 0 to 360°. They reported a side mode suppression ratio (SMSR) of 2.7 dB. An experimental demonstration of beam steering with a metasurface array operating at 1.3 μm was also recently presented [15]. The metasurface unit cell consisted of a metal–dielectric–oxide structure with indium tin oxide as an active layer, modulated by using top fan-out electrodes. The metasurface array was pixelated in two-dimensions and exhibited a phase change of 137°, allowing for two-dimensional beam steering to an angle of 7.3°. The side mode suppression ratio (SMSR) ranges from −9 to 6 dB.

Here we focus on the development of a reflectarray following a nanoscale pixel design based on a gold dipole nanoantenna that enables phase control in reflection, explored theoretically in previous work [7]. We demonstrate phase tuning with a constant magnitude of reflection and the absence of secondary lobes due to the sub-wavelength dimensions of the pixels. In the forthcoming sections, we describe and discuss the device concept, our fabrication techniques and our test set-up, and we present measurements compared with theoretical results based on electromagnetic and quantum carrier models of the structure. We give brief conclusions in the last section of the paper.

## 2 Description and operation of the reflectarray

**Figure 1:**
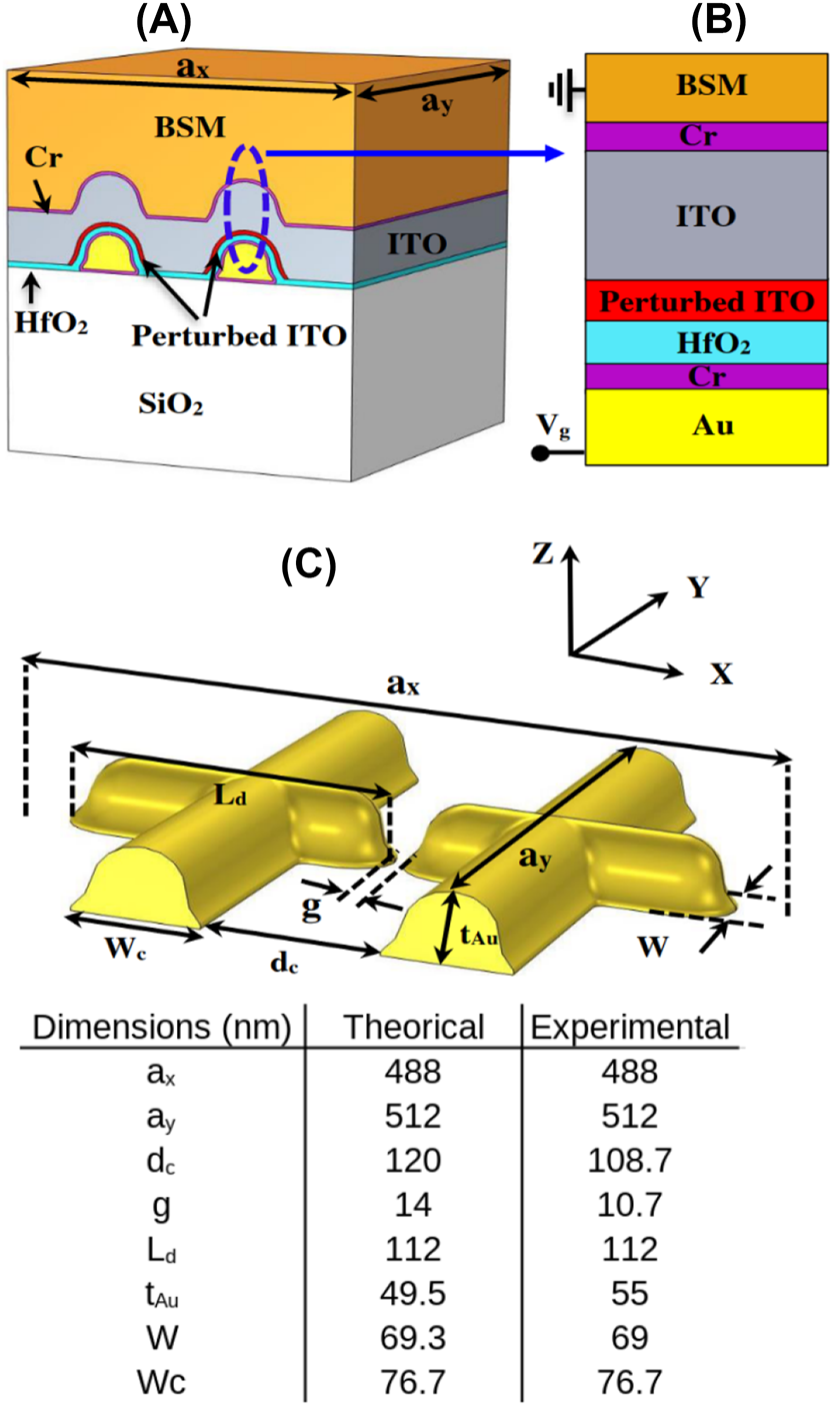
Sketch of the sub-wavelength unit cell used in the reflectarray, including the dimensions used in the theoretical model of the experimentally realised structure. (A) Panoramic view of the complete material stack of the unit cell, (B) 1D MOS capacitor enabling electrical control of the optical performance of the pixel, and (C) panoramic view of gold dipole nanoantennas along with connectors.

Figure 1(A) illustrates the plasmonic unit cell (pixel) of dimensions *a*_*x*_ by *a*_*y*_ employed in our reflectarray. The unit cell exhibits periodicity along both the *x* and *y* directions. The pixel design comprises a gold dipole nanoantenna, formed by two rods of length *L*_*d*_, width *W*, and thickness *t*_*Au*_. These rods are separated by a gap *g* and intersected by two perpendicular gold connectors of width *W*_*c*_, positioned at an edge-to-edge distance of *d*_*c*_ from each other, as shown in Figure 1(C). The dimensions of the dipole and connectors within the plasmonic unit cell are presented in the table inset in Figure 1.

Within this unit cell, a metal dipole nanoantenna is positioned with its long axis aligned in the *x*-direction. This configuration enables the structure to resonate in its fundamental mode at the desired wavelength when excited by a plane wave or beam that is polarized in the *x*-direction and propagating in the *z*-direction (upward through the SiO_2_ substrate). In the case of this particular polarization (or any polarization in the *z* − *x* plane), electrical contacts can be strategically placed along each segment of the antenna in the *y*-direction as shown in Figure 1. This arrangement produces a nanoantenna array that can be addressed column-wise when viewing the *x* − *y* plane (*cf.* Figure 4(H)). Thus, the structure is designed to serve not only as an antenna but also as device electrodes.

The gold antennas and contacts are supported by a glass (fused silica) substrate. They were deposited on a 3 nm layer of chromium to ensure adherence, as shown in magenta in Figure 1(A) and (B). Additionally, a 3 nm layer of chromium was deposited onto the gold antennas to facilitate the subsequent oxide layer. Following this, the structure is further conformally coated by a 7 nm layer of insulating oxide (hafnia, HfO_2_), followed by a 75 nm thick layer of indium tin oxide (ITO) as the semiconductor, and finally by a ground Ohmic contact designated as the backside metal (BSM) also functioning as an optical mirror, comprised of the metal stack 3 nm Cr + 50 nm Au + 500 nm Cu + 50 nm Pt. This layer arrangement forms electrically a MOS (metal oxide semiconductor) capacitor structure, operating optically as a resonant nanoantenna on an insulator/ITO stack, on a mirror.

The device is constructed such that ITO fills the gap between the antenna arms, which is where the optical fields are enhanced and the structure is most sensitive. When the MOS structure is driven into strong accumulation, a high electron concentration layer is formed within a confined area approximately 4 nm away from the interface of the ITO and the insulator. We refer to the accumulation layer as the perturbed layer in ITO, indicated in red in Figure 1(B). Furthermore, ITO undergoes a transition through epsilon-near-zero (ENZ) in this layer as accumulation takes place, resulting in a significant perturbation of the antenna resonance, including its phase.

The antenna unit cells (pixels), of dimensions *a*_*x*_ by *a*_*y*_, are distributed in a periodic fashion over a designated area measuring 10 × 10 μm^2^, centered within an electrical fan-out structure to contact an array column-wise. The selection of pixel periodicity is crucial, and is chosen small enough to satisfy the condition *a*_*x*_, *a*_*y*_ ≤ *λ*/2, where *λ* represents the wavelength of operation in the medium through which light is incident on the nanoantennas (glass substrate). By respecting this condition, the arrangement ensures the absence of grating diffraction orders in reflection.

## 3 Materials and methods

### 3.1 Fabrication process

The fabrication process employed three distinct lithography techniques. A trilayer contact photolithography process was employed to create relatively large features via lift-off, such as a fan-out structure, contact fingers and pads, and the backside metallization. For specific details of this fabrication process, refer to [16]. This technique provides better resolution than conventional bilayer photolithography lift-off processes. A traditional single-layer photolithography process was used to create a wet etch mask to define ITO regions. A helium ion beam lithography technique was utilized to fabricate the smallest features via lift-off, such as the nano-antennas and its crossing contacts to fan-out structures. This technique provides better resolution than e-beam lithography while minimising proximity effects. A detailed description of our helium ion beam lithography process can be found in [17].

**Figure 2:**
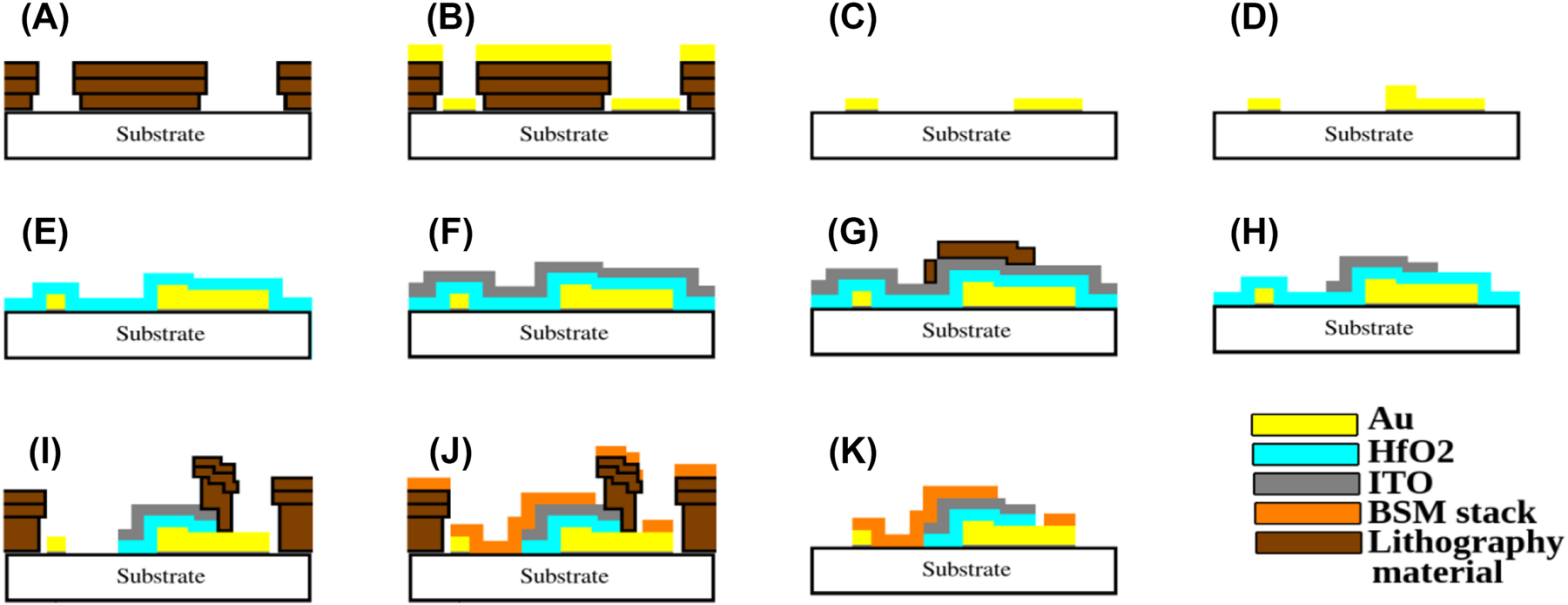
Fabrication process flow. (A) Trilayer photolithography stack to define FML on fused silica substrate; (B) FML deposition by evaporation (3 nm Cr + 28 nm Au); (C) lift-off; (D) fabrication of nanoantenna arrays by helium ion beam lithography, metal evaporation, and lift-off (3 nm Cr + 55 nm Au + 3 nm Cr); (E) atomic layer deposition of 7 nm thick hafnia layer; (F) sputter deposition of 75 nm thick ITO layer followed by annealing at 350 °C in O_2_; (G) wet etch mask application; (H) ITO wet etch in bath of TE-100 (Transene); (I) trilayer photolithography stack to define BSM, followed by removal of exposed hafnia via reactive ion etching (RIE); (J) BSM deposition by evaporation (3 nm Cr + 50 nm Au + 500 nm Cu + 50 nm Pt); (K) lift-off, revealing completed device.

The complete fabrication flow is depicted in Figure 2. The fabrication process begins with the patterning of the first metal layer (FML) on a fused silica substrate using a trilayer photolithography process [16], as illustrated in Figure 2(A). The FML was deposited via evaporation as the metal stack 3 nm Cr + 28 nm Au + 2 nm Cr. The top layer of Cr was added to improve adhesion for the subsequent oxide application, as illustrated in Figure 2(A) and (B). It was then lifted-off such that the desired features remained on the substrate, as shown in Figure 2(C). The metal features defined in this step include alignment marks for subsequent process steps and to facilitate experimental characterization, a fan-out structure, contact pads to connect to a nanoantenna array, and various process control structures. Then, the nanoantenna arrays and crossing contacts were fabricated using a helium ion beam lithography and liftoff process [17], as the metal stack 3 nm Cr + 55 nm Au + 3 nm Cr, as shown in Figure 2(D). Electron beam lithography was employed to expose alignment marks into the resist enabling helium ion beam visibility and the overlay of the nanoantennas onto the FML. This was necessary since the FML cannot be imaged through PMMA with a helium ion beam unlike an electron beam. The crossing contacts were aligned and overlapped with the fan out features on the FML to ensure electrical continuity of the nanoantennas to the contact pads. Then we used an atomic layer deposition (ALD) process to conformally deposit a 7 nm thick layer of hafnia, as shown in Figure 2(E). This was followed by the deposition of a 75 nm thick ITO layer via sputtering, which was then annealed at 350 °C in O_2_, as shown in Figure 2(F). A wet etch mask was then defined photolithographically, as shown in Figure 2(G), the ITO layer was etched in a bath of TE-100 etchant (Transene Inc.), and the photoresist mask was stripped, as shown in Figure 2(H). The ITO patterns thus formed consisted of disks centered above the nanoantenna arrays and their crossing contacts. Then we defined the BSM using a trilayer photolithograpy process [16], and we removed the exposed hafnia layer using a reactive ion etching (RIE) process prior to BSM deposition, as shown in Figure 2(I). The BSM was then deposited via evaporation as the metal stack 3 nm Cr + 50 nm Au + 500 nm Cu + 50 nm Pt, as shown in Figure 2(J), followed by lift-off to form complete structures, as shown in Figure 2(K). The BSM pattern consisted of circular patches on the ITO disks defining the Ohmic contacts and mirrors thereon, and overlapped metal on the fan-out and pad structures to reduce the resistance and facilitate probing.

Witness samples were included during the deposition of hafnia and ITO for the purpose of evaluating the films independently. The hafnia layer had a density of 9.61268 g/cm^3^ and a thickness of 7.76 ± 0.258 nm, confirmed using X-ray reflectometry (XRR). The electronic properties of the ITO layer were determined using an Ecopia HMS-3000 Hall effect measurement system in Van der Pauw configuration. The optical properties of the deposited ITO and hafnia layers were extracted from ellipsometry measurements using a spectroscopic ellipsometer (UVISEL Plus, Horiba Scientific). See Section 1 under Supplementary Material for details.

### 3.2 Electro-optic test set-up and characterization techniques

**Figure 3:**
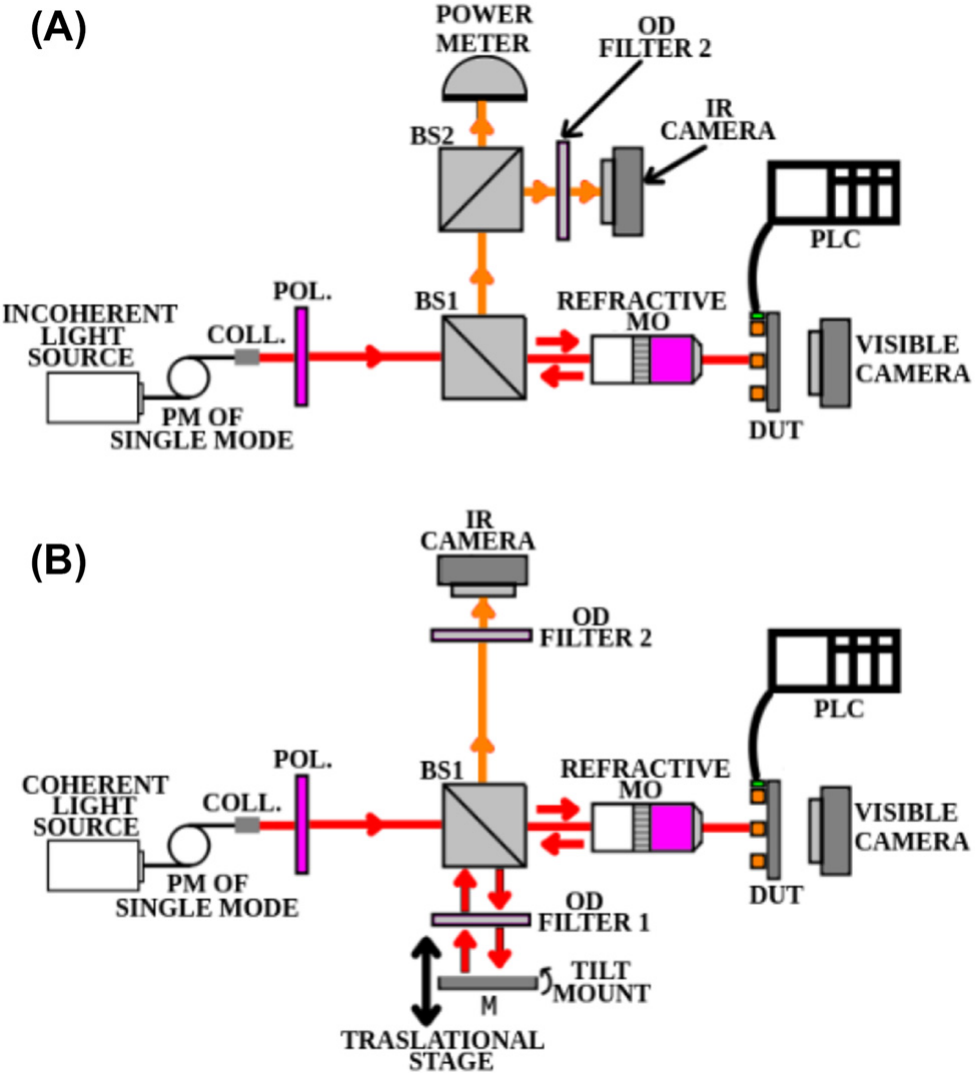
Confocal microscope for measuring (A) the magnitude of the reflection coefficient |Γ(*V*)|, and (B) its relative phase *ϕ*(*V*).

To investigate the reflectarray’s electro-optical performance, we used two custom-built confocal microscopes. Figure 3(A) shows the setup used to measure the magnitude of the reflection coefficient |Γ(*V*)|, and Figure 3(B) shows the setup used to measure the relative phase shift *ϕ*(*V*), both at normal incidence and at bias voltage *V*. In both setups, the input light beam was delivered through a polarization-maintaining single-mode optical fiber (PMOF) and collimated using a fiber collimation package (F220APC-1550, Thorlabs). A polarizer (LPNIR100, Thorlabs) was inserted to fix the polarization orientation parallel to the nano-antennas. The incident beam was focused onto the reflectarray using a plan apochromatic NIR objective (IR18091161 50×, NA = 0.42, BoliOptics). The reflected beam was redirected by beamsplitter BS1 (BSW29, Thorlabs) to the measurement instruments. For the setup shown in Figure 3(A), beamsplitter BS2 allowed monitoring the beam spot using an IR camera (Micronviewer 7290A, Electrophysics) while taking power measurements (81525A, Agilent/HP). For the setup shown in Figure 3(B), beamsplitter BS1 and mirror M produced a reference beam used to interfere with the beam reflected by the reflectarray on the IR camera (this setup effectively integrates a Michelson interferometer at the input of the confocal microscope).

We used two different light sources for our measurements. An incoherent light source was used to measure the magnitude of the reflection coefficient (Figure 3(A)), whereas a coherent light source was used to measure the relative phase shift (Figure 3(B)). The incoherent light was delivered by a super-continuum white light source (SuperK Extreme, NKT Photonics) with an acousto-optic tunable filter (SuperK SELECT, NKT Photonics), whereas the coherent light was delivered by a tunable laser (TUNICS-Plus CL, 1510 nm–1640 nm, Photonetics).

The sub-wavelength reflectarray was designed to reflect the incident beam without secondary lobes (which would be apparent in our set-up on images captured by the IR camera). It is desirable for the incident beam spot to be smaller than the area of the reflectarray (10 × 10 μm^2^) to minimize diffraction from its edges [18].

To ensure this, the beam spot diameter was measured for both of our sources (super-continuum and the tunable laser) using the knife-edge method [19], resulting in a 8.5 μm diameter, fulfilling this requirement.

To apply bias voltages to the device under test (DUT), we used a custom probe card (Accuprobe) to simultaneously contact the 42 pads of a DUT: 2 pads connected in common to the device’s ground, and 40 pads connected columnwise to the nanoantenna array to apply the gate voltages. Alignment of the probe card to a DUT was facilitated by a miniaturised microscope camera positioned on the backside of the DUT, as depicted in Figure 3. We used a programmable logic controller (PLC) (Productivity2000, Automation Direct) with analog output modules (P2-08DA-2, 8 outputs, Automation Direct) and two 16 bit digital to analog converters (P2-16DA-2, 32 outputs, Automation Direct) to apply a bias voltage individually to every pad of the DUT. Each analog output had a voltage resolution of 0.305 V/count, with a maximum possible count of 65536 (16 bits), and a maximum/minimum DC output voltage of ±10 V. The maximum available current per output was 10 mA.

We measured the magnitude of the relative reflection coefficient |Γ(*V*)| at different bias voltages using the setup shown in Figure 3(A). |Γ(*V*)| was taken as $\sqrt{P_{m}/P_{\mathrm{sub}}}$ , where *P*_*m*_ is the power reflected by the reflectarray, and *P*_*sub*_ is the power reflected by a nearby region of the substrate without nanoantennas but coated with hafnia, ITO and the backside Au mirror. *P*_*sub*_ can be used as a reference power because the hafnia and ITO layers are very thin and the backside mirror is a good approximation to a perfect mirror at the wavelengths of interest. Optical density filter 2 (OD 2) was adjusted to avoid saturating the IR camera. We kept the applied bias voltage equal and constant on all pads while sweeping the wavelength.

The phase shift measurement relied on the intensity change resulting from the interference between the beam reflected by the device under test (DUT) and the reference beam reflected by mirror M (Figure 3(B)). First we model the overlapping beams as two interfering waves. We write the wave representing the reference beam as *w*_1_(*z*, *t*) = *A* cos(*kz* − *ωt*), and the wave representing the beam reflected by the reflectarray as *w*_2_(*z*, *t*) = |Γ(*V*)|*B* cos(*kz* − *ωt* + *ϕ*(*V*)). Setting *z* = 0 as the camera location and *t* = 0 yields *w*_1_ = *A* and *w*_2_ = |Γ(*V*)|*B* cos(*ϕ*(*V*)). The intensity of the interference of the beams on the camera can be expressed

(1) $$\begin{array}{ll} I\left(V\right) & ={\left[A+|\Gamma \left(V\right)|B\cos\left(\phi \left(V\right)\right)\right]}^{2}\\ & =A^{2}+2AB|\Gamma \left(V\right)|\cos\left(\phi \left(V\right)\right)\\ & \;+|\Gamma \left(V\right){|}^{2}B^{2}\cos^{2}\left(\phi \left(V\right)\right), \end{array}$$

Initially, the bias voltage was set to zero. To achieve equal intensity for the reflected and reference beams on the camera, OD 1 and OD 2 were carefully adjusted. This involved individually blocking one beam’s optical path while adjusting the optical density (OD) filter of the other.

To align both beams on the camera, the tilt mount of mirror M was adjusted until the position of the reference beam almost perfectly overlapped with the reflected beam. Subsequently, the translation stage of mirror M was fine-tuned to achieve total constructive interference. The intensity *I*(0) was captured under the conditions that the intensity of individual beams *w*_1_ and *w*_2_ were the same and constructively interfering (*A* = |Γ(0)|*B* and cos(*ϕ*(0)) = 1). From equation (1) at *V* = 0 V, we deduced $A=\frac{\sqrt{I\left(0\right)}}{2}$ .

Then a non-zero and identical bias voltage was simultaneously applied to all pads of the device, and *I*(*V*) was measured. By assuming that the intensity change in the resulting interference patterns was solely due to the phase shift or that the magnitude of the relative reflection coefficient at a given wavelength is almost constant (|Γ(*V*)| ≈ |Γ(0)|), we solved cos(*ϕ*(*V*)) in Eq. (1) using the quadratic formula:

(2) $$0=\left(A^{2}-I\left(V\right)\right)+2A^{2}\cos\left(\phi \left(V\right)\right)+A^{2}\cos^{2}\left(\phi \left(V\right)\right).$$

The solutions of Eq. (2) are:

(3) $$\cos\left(\phi \left(V\right)\right)=\frac{-A+\sqrt{I\left(V\right)}}{A}$$

(4) $$\cos\left(\phi \left(V\right)\right)=\frac{-A-\sqrt{I\left(V\right)}}{A}$$

where the second solution is dropped for producing values out of range of the cosine function. Isolating the phase shift *ϕ*(*V*) from Eq. (3), yields:

(5) $$\phi \left(V\right)=\cos^{-1}\left(\frac{\sqrt{I\left(V\right)}-A}{A}\right),$$

where *I*(*V*) has been measured and *A* determined as described above. See Section S2 under Supplementary Material for a an example calculation of the phase shift from the raw data.

### 3.3 Capacitance versus voltage (C–V) measurements

We measured individual capacitance–voltage (C–V) characteristics for every nanoantenna column using an HP 428a LCR meter, at an AC voltage of ±30 mV and a frequency of 10 kHz. The DC bias voltage was increased from −2 V in steps of 0.05 V until the breakdown voltage (BDV) was reached in accumulation. The C–V measurements were performed after the electro-optic measurements because the capacitor structures become compromised when the BDV is reached.

From the C–V characteristics, we determined the dielectric constant (DC relative permittivity) of hafnia using:

(6) $$\epsilon_{ox}=C_{\max}\frac{t_{ox}}{A_{p}\epsilon_{0}},$$

where *C*_max_ is the maximum capacitance measured in accumulation, *t*_*ox*_ is the thickness of the hafnia layer, *A*_*p*_ is the capacitor area probed by pad *p*, and *ϵ*_0_ is the vacuum permittivity. The breakdown field (BDF) in MV/cm was calculated using

(7) $$\text{BDF}=\text{BDV}\times 10/t_{ox,nm},$$

where the breakdown voltage BDV is in *V* and *t*_*ox*,*nm*_ is the thickness of the hafnia layer in nm.

### 3.4 Electrostatic modelling

Classical models such conventional drift-diffusion (CDD) [20], [21] are widely used to compute the voltage-dependent carrier density profile in the semiconductor of MOS capacitors. However, when the MOS structure is incorporated into a plasmonic structure or metasurface, and the semiconductor is an epsilon-near-zero medium (such as ITO), then effects such as quantum confinement, quantum tunneling, quantum compressibility and electron diffraction/interference need to be considered. These factors are crucial to accurately compute the profile of the carrier density [22], [23], [24]. The details of the profile significantly impact the accuracy of the resulting permittivity distribution, particularly regarding the number and location of epsilon-near-zero points [25]. Here, we used self-consistent Schrödinger–Poisson (SP) coupling to compute the carrier density profile in ITO under accumulation [25]. This model is theoretically more rigorous than CDD because it takes the aforementioned quantum effects into account. We used the semiconductor physics node available under the semiconductor module of COMSOL 6.1, and the Schrödinger–Poisson interface with the equilibrium study [26].

In materials like ITO, which are sensitive to local refractive index perturbations, the SP model shows significant deviations from classical models in predicting carrier density under accumulation. The conventional drift-diffusion (CDD) model [25] predicts the highest carrier density at the HfO2-ITO interface, decreasing exponentially from the boundary. This prediction contrasts with the SP model, which suggests a lower density at the interface and a peak within the ITO. The CDD model, treating carriers as a classical free electron gas, overlooks quantum effects such as quantization, confinement, and diffraction/interference, which are accounted for in the SP model. These quantum effects lead to findings like the reduced carrier density at the HfO2-ITO interface due to quantum confinement and oscillatory profiles at higher gate voltages. At sufficiently high voltages, the SP model predicts the carrier density in ITO crossing two ENZ points, contrasting with the single ENZ point by the CDD model. This discrepancy significantly affects the permittivity profile and the optical response of the material [25].

The Poisson and Schrödinger equations are coupled second-order nonlinear differential equations, and solving this system is nontrivial, requiring an iterative approach. To start the iterations, we computed the potential distribution throughout the 1D MOS capacitor (Figure 1(B)) via the CDD and used it as an input to the SP model. Since the Cr is just 3 nm, we assumed that voltage is applied to the gold and ignored the Cr in electrostatic simulation. To obtain the potential distribution, we applied a gate voltage to the metal and grounded the ITO. The metal work function was set to 5.1 eV, whereas the electron affinity, bandgap energy, static relative permittivity, and doping concentration of the ITO were set to 4.8 eV, 2.8 eV, 9.1, and 2.65 × 10^20^ cm^−3^, respectively.

The potential distribution obtained from the CDD was then input into the SP model by updating the potential term therein. In the next step, the carrier density profile *N*(*d*) is computed using the sum of statistically weighted densities of the eigenfunctions obtained from the updated Schrödinger equation via [22], [25]

(8) $$N\left(d\right)=\underset{i}{\sum }W_{i}|\phi_{i}\left(d\right){|}^{2},$$

where *W*_*i*_ represents the weight factors, *ϕ*_*i*_ is the *i*th normalized eigenfunction associated with eigenenergy *E*_*i*_, and *d* denotes the distance from the oxide surface. Then, the new potential distribution is computed by solving Poisson’s equation using the updated charge distribution *N*(*d*) (Eq. (8)) in the charge density term therein. This procedure is repeated until the electrostatic potential distribution converges. A uniform mesh of step size 0.1 nm was used to discretize the structure. Further details on setting up the problem in COMSOL can be found elsewhere [25].

We then used the electron density distribution computed by the SP model *N*(*d*) in the Drude equation [27] to obtain the spatially-dependent relative permittivity in the perturbed ITO:

(9) $$\epsilon \left(d,\omega \right)=\epsilon_{\infty }-\frac{\omega_{D}{\left(d\right)}^{2}}{\omega^{2}+i\gamma_{D}\omega },$$

where *ϵ*_∞_ is the high-frequency relative permittivity and *γ*_*D*_ is the collision frequency, which were taken as 3.92 and 4.4 × 10^13^ rad/s, respectively [25]. *ω*_*D*_(*d*) is the spatially-dependent plasma frequency of the ITO obtained via

(10) $$\omega_{D}\left(d\right)=\sqrt{\frac{N\left(d\right)}{n_{b}}}\omega_{D,b}$$

where *N*(*d*) is obtained for an applied bias voltage of interest. *ω*_*D*,*b*_ is the bulk plasma frequency of ITO and was fixed to 1.55 × 10^15^ rad/s [25].

### 3.5 Optical modelling

To investigate the gate-tunable optical response of the reflectarray, we used the relative permittivity distribution in ITO computed for different applied gate voltages as described in Subsection 3.4 as well as the experimentally-determined dimensions of the nanoantenna array (Figure 1). The carrier density in the perturbed layer is a function of distance from the hafnia-ITO interface, so we projected the 1D profile computed for the 1D MOS (Figure 1(B), red region) over the 3D unit cell (Figure 1(A), red region).

We used the electromagnetic waves, frequency domain interface of the wave optics module in COMSOL 6.1 for the optical modelling [26], and we computed the complex reflection coefficient of a unit cell versus wavelength at various applied bias voltages. The wave equations derived from Maxwell’s equations were solved by applying material properties and boundary conditions [28]:

(11) $$\nabla \times \nabla \times E\left(x,y,z\right)-k^{2}\epsilon_{r}\left(x,y,z\right)\mu_{r}E\left(x,y,z\right)=0$$

(12) $$\nabla \times \left(\frac{1}{\epsilon_{r}\left(x,y,z\right)}\nabla \times H\left(x,y,z\right)\right)-k^{2}\mu_{r}H\left(x,y,z\right)=0,$$

where *μ*_*r*_ is the relative permeability (set to 1), *ϵ*_*r*_(*x*, *y*, *z*) is the relative permittivity distribution describing the structure, *k* is the free-space wavenumber, and **E** and **H** are the electric and magnetic field vectors.

The structure varies along the *z*-axis and is periodic along the *x* and *y* directions (Figure 1). The propagation and electric field directions of the incident light were taken along the +*z* and *x* directions, respectively. Periodic boundary conditions were applied on the *x* and *y* boundaries, and a source port was placed along the bottom to excite the structure and record the reflected wave (the excitation propagates through the SiO_2_ substrate, in accord with the experimental situation). The modeling domain in the *z* direction was truncated and two perfectly matched layers (PMLs) were added to the top and bottom of the unit cell to absorb the incident waves.

In our optical model, the refractive index of hafnia was taken from experimental ellipsometry data (see Section S1), the relative permittivity of Au and Cr were taken from Johnson and Christy [29] and Rakić et al. [30], respectively, and the voltage-dependent relative permittivity distribution of the ITO – obtained from our electrostatic calculations in Subsection 4.2 – was used. We used mesh mapping along the top boundaries of the perturbed region in ITO and swept it throughout the 3D domain of this region in a step of 0.1 nm. A tetrahedral mesh was used for the remaining domains.

## 4 Results and discussion

### 4.1 Fabrication

**Figure 4:**
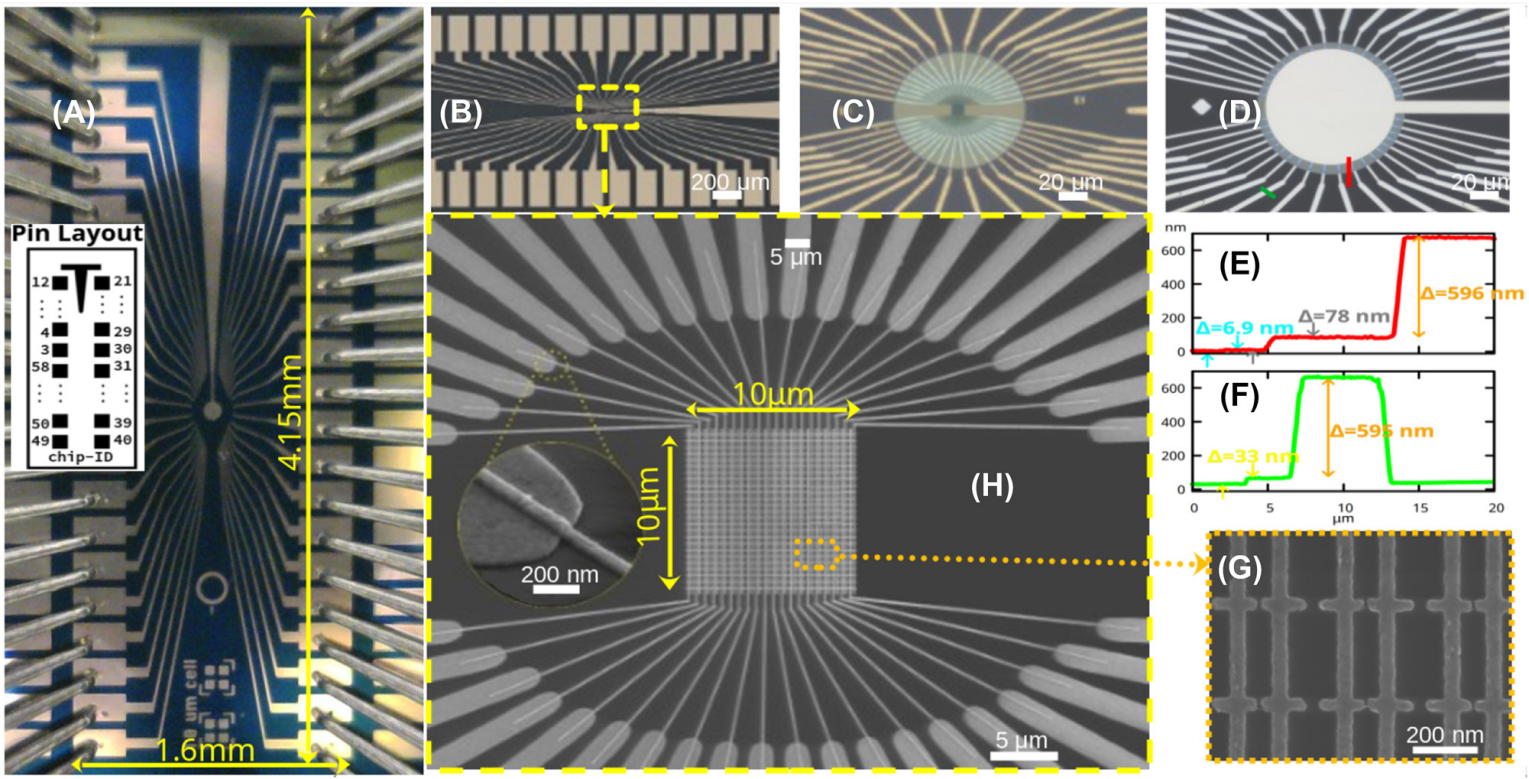
Images of fabricated devices. (A) Microscope image of complete device with pins landed on contact pads. Inset: Pad layout of the device and notation. The same layout was used for the pins of the probe card. (B) Microscope image (50×) of the first metal layer (FML) and the nanoantenna array. (C) Microscope image (500×) of the device center showing the fan-out fingers reaching beneath the circular ITO patch. (D) Microscope image (500×) of the device center after backside metal deposition. (E) and (F) AFM scans along two locations showing step changes in the material stack due to hafnia, ITO and backside metal (scan taken along red line sketched in part (D)), and step changes in the material stack due to the first metal layer and the backside metal layer (scan taken along green line sketched in part (D)). (H) HIM image at low magnification revealing the nanoantenna array and the nanoscale fan-out fingers connecting the array to the large-scale fan-out fingers on the FML (high-magnification inset shows good step coverage of these fingers). (G) HIM image at high magnification of a few unit cells of the nanoantenna array.

Completed devices produced by the fabrication process described in Subsection 3.1 are shown in Figure 4, highlighting various stages in the fabrication flow. Figure 4(A) shows a completed device being probed, and the inset depicts the pad, pin layout, and numbering. Figure 4(B) shows the device after FML formation and fabrication of the nanoantennas in the central area of the device. Figure 4(C) shows the central area of the device after hafnia and ITO deposition/lift-off (central circular patch). In Figure 4(D), the same area is shown after BSM formation, which is the final step in the fabrication flow. Figure 4(E) and (F) present AFM scans taken along the red and green lines sketched on Figure 4(D). Figure 4(E) shows step changes in the material stack corresponding to the 6.9 nm thick hafnia layer, the 78 nm thick ITO patch, and the 596 nm thick BSM, whereas Figure 4(F) shows the step changes in the material stack corresponding to the 33 nm thick FLM and 595 nm thick BSM. In Figure 4(H), a low-magnification helium ion microscope (HIM) image reveals the nanoscale fan-out structure connecting the columns of the nanoantenna array to the large-scale fan-out structure on the FML. The high-magnification inset shows good step coverage of these fingers. Figure 4(G) is gives a high-magnification HIM image of a nanoantenna array, from which feature dimensions are measured and reported in the table inset to Figure 1.

### 4.2 Electrostatic performance

**Figure 5:**
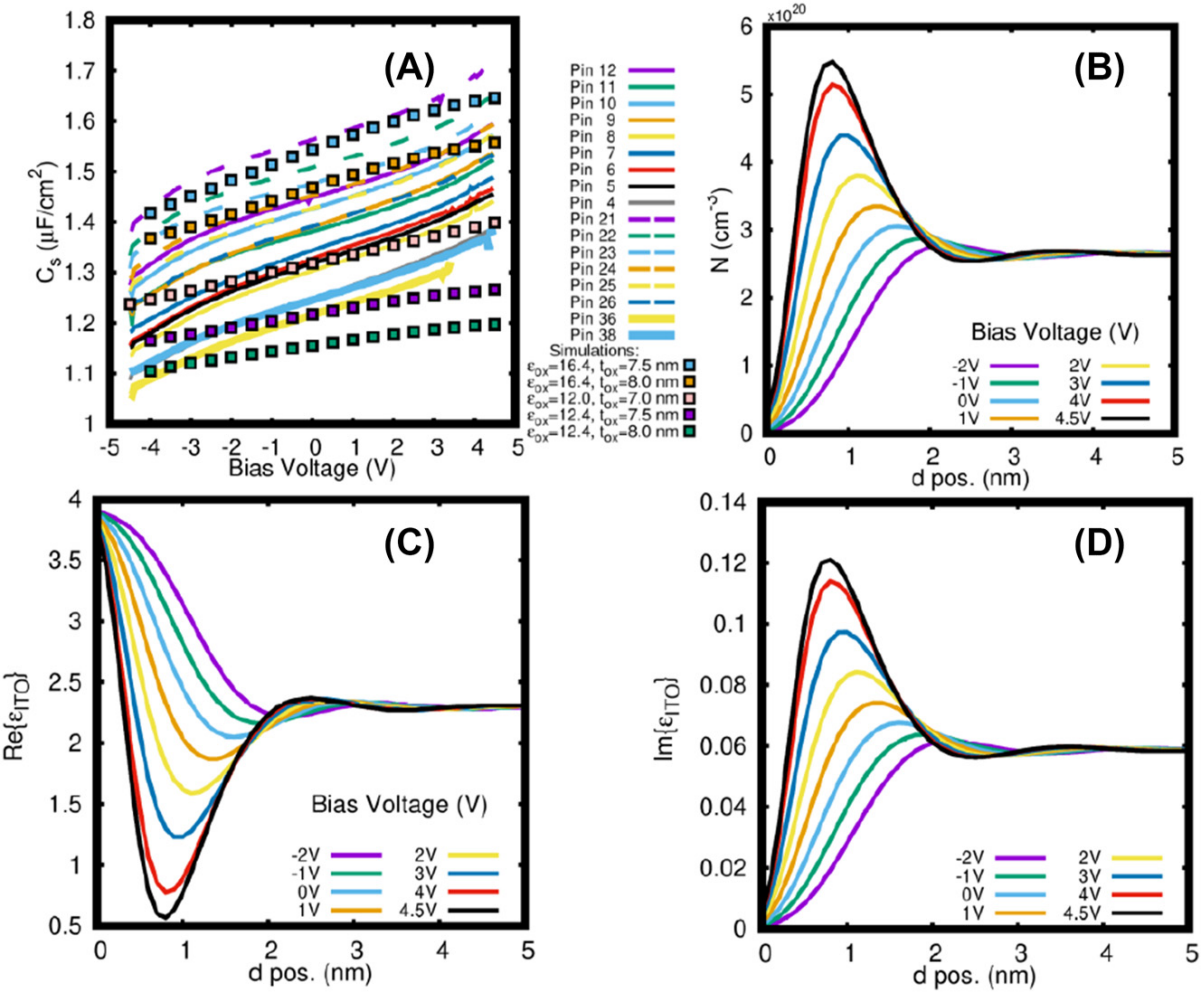
Electrostatic performance of sub-wavelength reflectarray. (A) Capacitance per unit area versus applied voltage (C–V curves), measured for several device columns, identified by pad and pin number. The computed C–V curve is also shown for comparison. (B) Computed carrier density profile from the hafnia-ITO interface into ITO for different bias voltages. (C) And (D) corresponding computed real and imaginary parts of the relative permittivity distribution into ITO.

In Figure 5(A), the graph depicts the computed capacitance per unit area plotted against the voltage applied to the sub-wavelength reflectarray, with the data point represented by colored squares. The computations involved three different values for the DC relative permittivity of hafnia, *ϵ*_*ox*_ = 12, 12.4 and 16.4, combined with three thickness values, *t*_*ox*_ = 7, 7.5 and 8 nm. These values were chosen to encompass the measured experimental C–V characteristics, as discussed subsequently in Section 4.4. Specifically, the combination *ϵ*_*ox*_ = 12 and *t*_*ox*_ = 7 nm generates theoretical C–V characteristics that are representative of the average experimental measurements. The C–V theoretical result was obtained by taking the derivative of the charge per unit area in ITO relative to the applied voltage, the former computed from the associated carrier density profiles. The corresponding measured C–V characteristics plotted on Figure 5(A) will be discussed in Subsection 4.4.

Figure 5(B) shows the electron carrier density profiles for *ϵ*_*ox*_ = 12 and *t*_*ox*_ = 7 nm. The electron carrier density was calculated inside the perturbed region versus distance from the hafnia-ITO interface for the gate voltages listed in the legend, computed using the SP model. It is clear that the perturbation extends to about 4 nm into the ITO from the hafnia-ITO interface (*z* = 0). For each applied voltage, a reduction in the carrier density is noted at the hafnia-ITO interface due to quantization of the electron states thereon. At gate voltages from −2 to 0 V the perturbed region is in depletion. By increasing the gate voltage, the carrier density increases, and the region transitions from depletion to accumulation at 1V. As noted in Figure 5(B), for positive voltages, the lower carrier density at the interface is followed by a clear maximum inside ITO, which grows and shifts toward the hafnia-ITO interface with increasing voltage and electron accumulation. The observed oscillatory behavior in the carrier density profile is due to electron wavefunction diffraction and interference [31].

Figure 5(C) and (D) show the real and imaginary parts of the relative permittivity of ITO, respectively, computed by inserting the electron carrier density profile shown in Figure 5(B) into the Drude equation (Eqs. (9) and (10)), at the free-space operating wavelength of *λ* = 1560 nm. As noted in Figure 5(C) and (D), the real and imaginary parts of the permittivity approach 4 and 0, respectively, at the hafnia-ITO interface for all gate voltages, due to electron depletion that develops thereon (Figure 5(B)), driven by the quantization of the electron states. A dip (bump) is then clearly noted in the real (imaginary) part of the permittivity at bias voltages ≥0 V. This dip (bump) occurs at the maximum of the carrier density in ITO (Figure 5(B)) and reduces (increases) with increasing gate voltage. The electric fields become localised and enhanced in regions where the relative permittivity of ITO becomes small [25].

The permittivity of ITO experiences perturbation through the carrier refraction effect induced in the material (recall that the ITO is the semiconductor in MOS capacitors). Voltage gating facilitated by metallic connectors induces a carrier density perturbation over a thin ITO region near the nanoantenna under accumulation and depletion. During accumulation, the carrier density increases, leading to a blue-shift of the epsilon-near-zero wavelength (*λ*_*ENZ*_) in the perturbed ITO region. The local changes in carrier density within the thin ITO layer, induced by accumulation or depletion in the MOS capacitor, lead to a local variation in permittivity due to the carrier refraction effect. As this perturbed ITO layer is situated in the enhanced field region near the plasmonic nanostructure, its refractive index variation alters the resonance of the nanoantenna, thereby influencing the reflection properties of the reflectarray, particularly its phase shift.

### 4.3 Electro-optical performance

**Figure 6:**
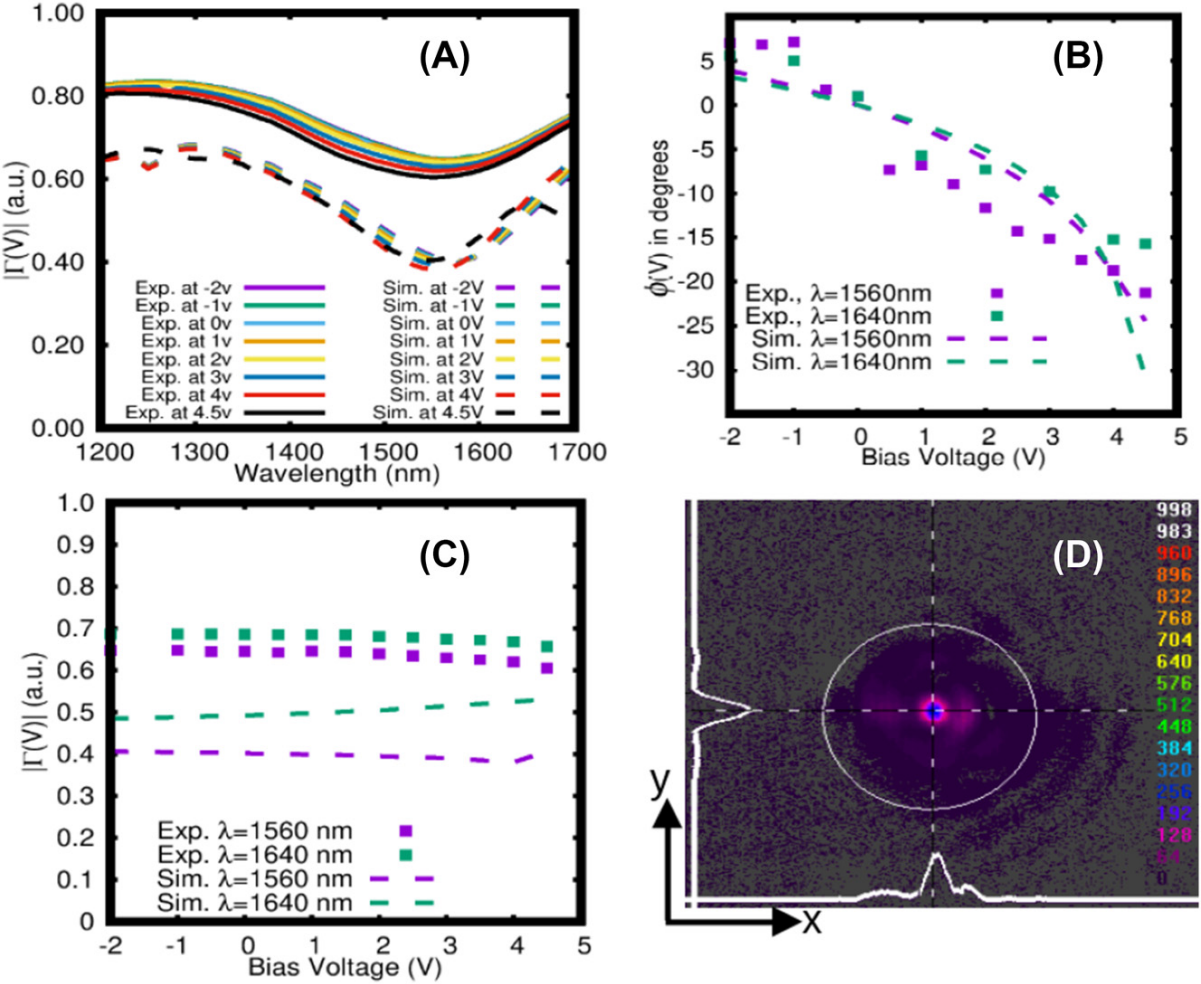
Electro-optical performance of sub-wavelength reflectarray. (A) Measured and computed |Γ| response for bias voltages between −2 and 4.5 V. (B) Measured and computed relative phase shift *ϕ* versus bias voltage at two operating wavelengths. (C) Measured and computed |Γ| versus bias voltage at two operating wavelengths. (D) Intensity contours of beam reflected from sub-wavelength reflectarray, captured by IR camera at zero bias voltage. Horizontal and vertical cross-sectional profiles are shown as the white plots.

Theoretical and experimental results summarising the electro-optical performance of the sub-wavelength reflectarray are presented in Figure 6. In Figure 6(A), the measured wavelength response of |Γ| exhibits a prominent dip at 1560 nm, indicating the resonance wavelength for strong coupling of the incident beam to the nanoantennas. Notably, |Γ| shows minimal variation across different applied bias voltages.

The experimental resonance wavelength closely matches the simulation results, thus validating the accuracy of the model. However, it is worth noting that the experimental |Γ| are slightly larger than the calculations. The difference between them is 0.15 off-resonance and 0.24 on-resonance. This difference is due to background light present during the measurements and imperfect coupling due to slight misalignment of the beam to the reflectarray.

Figure 6(B) gives the relative phase shift as a function of bias voltage for two wavelengths of operation, referenced to the phase shift at 0 V, calculated using Eq. (5). The wavelengths of interest are the resonant wavelength (1560 nm) and the maximum wavelength accessible on our tunable laser (1640 nm). On resonance (*λ* = 1560 nm), the relative phase shift was measured as 7.4° at −2 V and −22.8° at 4.5 V, resulting in a voltage-tuned phase range of 30°. Conversely, off-resonance (*λ* = 1640 nm), the voltage-tuned phase range is smaller, spanning 28.6° starting from 9.1° at −2 V and reaching −19.5° at 4.5 V.

Figure 6(C) gives the magnitude of the relative reflection coefficient as a function of bias voltage at the two selected wavelengths. The magnitude of the reflection coefficient remains flat over the bias voltage range investigated. Specifically, at the wavelength of 1560 nm, the experimental |Γ| exhibits a flatness of 4.9 %, about an average of 0.63. In comparison, the theoretical flatness is 4.4 %, about an average of 0.4. Likewise, at the wavelength of 1640 nm, the experimental |Γ| has a flatness of 3.3 % about an average of 0.68, whereas the theoretical flatness is 5.2 % about an average of 0.5. The flatness is defined as the ratio of the maximum deviation to the average value, multiplied by 100.

The insertion loss (IL) in dB of the reflectarray is defined as IL = −20 log_10_|Γ|. The experimental IL at 1560 nm is 4 dB, and at 1640 nm it is 3.35 dB. In contrast, the theoretical IL values are higher, 8 dB at 1560 nm and 6 dB at 1640 nm. The difference between them is attributed to background light present during the measurements and imperfect coupling due to slight misalignment of the beam to the reflectarray, as mentioned previously.

Figure 6(D) provides a visualization of the reflected beam spot from the reflectarray under no bias, including horizontal and vertical cross-sections along the center of the beam spot. Notably, the spot is very clean and absent of diffraction lobes. The vertical cross-section displays a well-formed Gaussian distribution, whereas the horizontal cross-section exhibits a weak diffraction pattern caused by scattering effects from the vertical edges of the reflectarray. The reflected beam maintained the same appearance as the bias voltage was varied over the range of Figure 6(C), including the weak diffraction pattern, owing to the flatness of the magnitude of the reflection coefficient.

These scattering effects are caused by the incident polarization and boundary conditions acting on the vertical edges of the reflectarray. Scattering from metallic edges occurs differently depending on whether the polarisation is hard or soft – the polarization is said to be hard when the boundary condition that applies is a Neumann condition, and soft for a Dirichlet condition [32]. The scattering pattern from a metallic edge is broader for the hard polarization than for the soft polarization, yielding an apparent larger beam diameter upon reflection. The incident light beam is linearly polarized and oriented horizontally, so *E*_*x*_ ≠ 0 and *E*_*y*_ = 0. As a consequence the electric field across the top and bottom (horizontal) edges of the reflectarray corresponds to the soft polarization, but across the right and left (vertical) edges it corresponds to the hard polarization yielding a broad scattering pattern along the *x*-axis. This undesired effect of diffraction from edges has been previously reported in other experimental work [33], [34].

### 4.4 Electrical characteristics of the hafnia layer

Figure 5(A) illustrates the voltage-dependent specific capacitance (C–V characteristics) measured for a reflectarray. The measurements exhibit repeatability and reveal distinct accumulation patterns in the capacitors of each column, identified by pin number. The raw capacitance measurements were offset on average by 0.0781 pF relative to the calibrated probes, leading to an overestimation of the specific capacitance by Δ*C*_*s*_ = 0.1 μF/cm^2^. This offset was subtracted from the data presented in Figure 5(A). More details on this point are given in Section S3 of the Supplementary document. The resulting C–V characteristics highlight variations in specific capacitance among the columns, suggesting discrepancies that should ideally converge into a single curve. These deviations are attributed to variances in the hafnia layer.

Analysis of the measured C–V data allows the dielectric constant (DC relative permittivity) of the hafnia layer to be extracted using Eq. (6), assuming a constant hafnia thickness of *t*_*ox*_ = 7.76 ± 0.258 nm (*cf.* Subsection 3.1, XRR measurements). The dielectric constant was calculated for each pin and ranged between *ϵ*_*ox*_ = 15.85 ± 0.511 for pin 21 and *ϵ*_*ox*_ = 12.43 ± 0.401 for pin 38, corresponding to the maximum and minimum capacitance values shown in Figure 5(A). These pins are positioned at the top and bottom of the device, respectively, indicating a position-dependent variation in the permittivity of the hafnia layer across the device.

The dielectric strength of the Hafnia layer was determined using Eq. (7), yielding a breakdown field (BDF) of 5.735 ± 0.2 MV/cm. The BDF reported in the literature for thin (ALD) hafnia is ≈10 MV/cm [35]. The low BDF value measured here for the hafnia layer prevents the MOS structures from reaching deep accumulation.

The non-uniformity observed in the hafnia layer, along with its low relative permittivity and BDF, suggests the presence of nanopores and a low density relative to bulk hafnia. Referring to the fabrication flow, we note that the metal stack of the nanoantennas, Figure 2(D), consists of a layer of Cr, followed by a layer of Au, capped by a thin layer of Cr, implying that the hafnia layer must nucleate on this surface. We suspect an insufficient surface density of hydroxyl (OH) functional groups on this metal stack, compromising hafnia nucleation during the initial cycles of the ALD process and resulting in reduced density of the film [36], [37]. Further work on improving the formation of hafnia on such metal stacks would be required.

### 4.5 Epsilon near zero in ITO

The deposited ITO layer exhibits an epsilon-near-zero (ENZ) state in the near-infrared (NIR) wavelength range, as confirmed by the analysis on witness samples (refer to Section S1 in the Supplementary Material for details). The device’s performance is significantly influenced by the ENZ wavelength of the ITO layer. This perturbation in ITO permittivity near the nanoantenna, induced by carrier refraction, is facilitated by voltage gating, allowing for carrier density modulation through accumulation and depletion. The increased carrier density leads to a blue-shift in *λ*_*ENZ*_ during accumulation impacts the reflectarray’s reflection properties.

The wavelength at which the refractive index of ITO becomes zero, *λ*_ENZ_, can be determined using the following equation:

(13) $$\lambda_{\text{ENZ}}=\frac{2\pi c_{0}\sqrt{\epsilon_{\infty }}}{\omega_{D,b}}$$

where *c*_0_ is the speed of light in vacuum, *ϵ*_∞_ denotes the dielectric constant of ITO, and *ω*_*D*,*b*_ corresponds to the Drude frequency for the bulk. Eq. (13) is derived from the Drude dispersion equation assuming that *γ*_*D*_ ≫ *ω*_*D*,*b*_, as shown in Eq. (2) in the Supplementary Information file.

To estimate *λ*_ENZ_ of the ITO layer in the reflectarray, we used the parameters obtained in Section 1 of the Supplementary Information file. Given that the carrier concentrations measured via the Hall effect align well with *ω*_*D*,*b*_ derived from the ellipsometry fittings, we calculated *λ*_ENZ_ using an average $\omega_{D}^{\prime }=\left(\omega_{D\text{,Hall}}+\omega_{D\text{,Ell}}\right)/2$ , where *ω*_*D*,Hall_ is the Drude frequency for the bulk calculated using Eq. (3) based on the carrier concentration measured via the Hall effect, and *ω*_*D*,Ell_ represents the Drude frequency for the bulk obtained from the ellipsometry fitting. Using Eq. (13), the anticipated range of *λ*_ENZ_ is from *λ*_ENZ_ = 1.91 μm to *λ*_ENZ_ = 2.1 μm. As the MOS structures enter the accumulation region, the carrier density in the perturbed ITO layer increases (Figure 5(B)), resulting in a blue-shift of *λ*_ENZ_ therein. If the breakdown field of hafnia is sufficiently high, *λ*_ENZ_ may transition through the resonance wavelength of the reflectarray, leading to a significant phase shift in the reflected beam [7]. Due to the low BDF of the hafnia layer here, it was not possible to apply a sufficiently large bias voltage for this regime to be attained.

### 4.6 Speed of operation

The response speed of the reflectarray is inherently limited by the time required for the MOS capacitors to reach full charge under accumulation, then to discharge when the bias voltage is reduced. The measured specific capacitances given in Figure 5(A), the corresponding areas (*cf.* Section S5 in the Supplementary Information file), and the minimum and maximum pin resistances determined to be *R*_pin_ = 1.8 kΩ and *R*_pin_ = 1.85 kΩ (*cf.* Section 4 in the Supplementary Information file), were considered in estimating the speed of operation. Based on these parameters, the time constant of the capacitors was calculated as *τ* = *R*_pin_ *C*_pin_, resulting in values ranging from *τ* = 1.98–2.5 ns, from which the maximum frequency of operation is determined to be 63 MHz (for *τ* = 2.5 ns).

## 5 Conclusions

We have successfully fabricated, experimentally characterized, and theoretically modelled a novel sub-wavelength reflectarray capable of varying the phase of the reflected beam without modifying its intensity or introducing scattering effects. The reflectarray pixels consisted of Au dipole nanoantennas integrated within a MOS capacitor structure. The pixels were fabricated on a fused Silica substrate, with a conformal coating of thin hafnia followed by layers of ITO and a Au backside mirror. By utilizing this MOS capacitor, we were able to modulate the carrier density of ITO at the interface with hafnia, thereby leading to the observed phase shift in the reflected beam. The reflectarray demonstrates excellent performance near 1550 nm, producing exclusively the desired specular reflected lobe. The reflectarray generated a maximum measured phase shift of about 30° at *λ* = 1560 nm, limited by the quality of our hafnia insulating film. The magnitude of reflection coefficient was about 0.5 and remained flat over the tuning range of the phase.

## Supplementary Material

Supplementary Material Details
