# Supplementary material for: Electrically tunable plasmonic metasurface as a matrix of nanoantennas
Source: Nanophotonics. 2024 Feb 27;13(6):901–13. doi: 10.1515/nanoph-2023-0796 (PMC11501110; doi:10.1515/nanoph-2023-0796)
Supplement: Supplementary file 1 — Supplementary Material Details [file j_nanoph-2023-0796_suppl_001.pdf]

Electrically tunable plasmonic metasurface as a matrix of nanoantennas: supplemental material

1 ITO and hafnia characterization

During the deposition process of the indium tin oxide (ITO) and hafnia layers, witness samples were created with the purpose of evaluate the hafnia and ITO layers. Sample SR83 had a target thickness of 79 nm of ITO deposited on fused Silica. Sample Hf4-227 consisted of a target thickness of 7 nm of hafnia deposited on fused Silica. Additionally, a bilayer sample (Hf3-227) was created, which consisted of a target thickness of 79 nm of ITO on top of 7 nm hafnia, both deposited on a fused Silica substrate.

The electronic properties of the ITO layer were analyzed using an Hall effect measurement system (HMS-3000, Ecopia) in the Van der Pauw configuration. The system measured the resistivity, carrier concentration, and mobility [1]. The Spring Clip Board Sample Mounting Boards (SPCB1, Ecopia) was used to hold and probe the sample, which had to be cut into squares of dimensions approximately 20 mm × 20 mm. Conductive ink drops were placed on the corners of the cut samples to ensure good electrical contact between the ITO surface and the needle probes. A magnetic field from a permanent magnet with a magnetic flux density of 0.55 T was used during the Hall effect measurements.

The optical characteristics of the deposited ITO and hafnia layers were extracted by taking ellipsometry measurements using a Spectroscopic Ellipsometer (UVISEL Plus, Horiba Scientific). This system uses a double modulation acquisition technology to measure the ellipsometry angles Ψ and Δ in reflection. The angle of incidence was kept fixed at 70°. The collected ellipsometry data were fitted to a layered model to determine the optical parameters of the ITO and hafnia layers. The algorithm used for the fitting was the Levenberg-Marquardt algorithm. The goodness of fit (GOF) was determined by:

$$\chi^2 = \min \sum_{i=1}^{n} \Big[\frac{(\Psi_{th} - \Psi_{exp})^2}{\Gamma_\Psi} + \frac{(\Delta_{th} - \Delta_{exp})^2}{\Gamma_\Delta} \Big] \tag{1}$$

where (Ψ_{th}, Δ_{th}) and (Ψ_{exp}, Δ_{exp}) are the theoretical and measured ellipsometry angles, and Γ_x is the standard deviation of each data point.

The refractive index of hafnia was modelled using the Cauchy dispersion model, $n(\lambda) = A + B\lambda^{-2} + C\lambda^{-4}$; $k = 0$. ITO was modelled using Drude dispersion model:

$$\epsilon(\omega) = \epsilon_\infty - \frac{\omega_{D,b}^2}{\omega^2 + i\gamma_D\omega}. \tag{2}$$

To analyze the transparent samples, we employed a backside reflection model. We used void as the substrate and added a 500 μm fused Silica layer. The total number of backside reflected beams was kept constant at 2. The percentage of first collected beam was set at 100% and the last collected beam was adjusted during the fitting process in order to minimize the GOF. The fused Silica layer was modeled via a multi-Sellmeier model, utilizing the bulk parameters reported in [2].

The ellipsometry fitting process began by extracting the optical parameters of hafnia from the Hf4-227 sample. Next, the Drude parameters for ITO were determined from sample SR83. Subsequently, the hafnia parameters obtained from the Hf4-227 sample were used to model the hafnia layer on

sample Hf3-227. For the ITO layer on the Hf3-227 sample, we used the Drude parameters obtained from the SR83 analysis as initial values in the fitting process.

To fit the ellipsometry data measured from Hf4-227, we used initial parameters for the Hafnia layer that correspond to bulk hafnia [3]. Specifically, $A = 1.875$, $B = 6.28 \times 10^3$ nm^2 and $C = 5.8 \times 10^8$ nm^4 in the Cauchy dispersion model. The thickness of the hafnia layer and the parameter A of the Cauchy dispersion model were used as fitting parameters, and the fitting wavelength range was set from 1000 to 2000 nm. We also included a roughness of 1 nm in the model, accounting for 50% air and 50% hafnia.

During the fitting for the Drude parameters of the ITO on sample SR83, we used initial parameter values for ITO as the average of values reported in [4–6]: $\epsilon_\infty = 4$, $\omega_{D,b} = 2 \times 10^{15}$ rad/sec, and $\gamma_D = 1.3 \times 10^{13}$ rad/sec. The thickness of the ITO layer was also a fitting parameter with initial value of 79 nm.

The model for the sample Hf3-227 consisted of a top layer of ITO over hafnia. The thickness of the ITO and the Drude parameters of the ITO layer were used as fitting parameters. The Hafnia layer was fixed at 7.2 nm which resulted from the fitting process on the sample Hf3-227. The initial thickness of the ITO layer was 79 nm and the initial parameters for the ITO were the resulted parametes from the fitting performend on sample SR83.

The resulted fitting process using the ellipsometry data from the witness samples were consistently very good. The GOF obtained was low across all cases studied. Specifically, for the model of sample Hf4-227 (hafnia on fused Silica), the value of χ^2 was 0.065, yielding a thickness of hafnia of 7.196 ± 0.862 nm and an A value of 1.872126 ± 0.0379. Similarly, for the model of sample SR83, the value of χ^2 was 0.074, and the resulting Drude parameters were $\epsilon_\infty = 4.0163 \pm 0.02$, $\omega_{D,b} = 1.7521 \times 10^{15} \pm 0.0105 \times 10^{15}$ rad/sec, and $\gamma_D = 0.1304 \times 10^{15} \pm 0.0032 \times 10^{15}$ rad/sec, with a ITO thickness of 79.16 ± 0.97 nm. Finally, for the model of sample hf3-227 (ITO on 7.2 nm hafnia on fused Silica), the value of χ^2 was 0.5, yielding an ITO thickness of 70 ± 2.6 nm and Drude parameters $\epsilon_\infty = 4.026 \pm 0.04152$, $\omega_{D,b} = 1.9515 \times 10^{15} \pm 0.015 \times 10^{15}$ rad/sec, and $\gamma_D = 0.1308 \times 10^{15} \pm 0.004 \times 10^{15}$ rad/sec.

The Hall effect measurements conducted on samples SR83 and Hf3-227 yielded the following results: for sample SR83, a carrier concentration of $n_b = -3.32 \times 10^{20}$ cm^{-3}, resistivity of $\rho = 0.428 \times 10^{-3}$ Ωcm, and mobility of $\mu = 43.93$ cm^2/Vs. For sample Hf3-227, a carrier concentration of $n_b = -4.433 \times 10^{20}$ cm^{-3}, resistivity of $\rho = 0.337 \times 10^{-3}$ Ωcm, and mobility of $\mu = 38.24$ cm^2/Vs were obtained.

The measured carrier concentration was in good agreement with the predicted Drude frequency $\omega_{D,b}$. This was confirmed by using

$$|n_{b,ell}| = \frac{\omega_{D,b}^2 \epsilon_0 m_n^*}{e^2 \times 10^6} \tag{3}$$

where $|n_{b,ell}|$ is the carrier concentration in cm^{-3}, $\omega_{D,b}$ is the Drude frequency in rad/sec obtained from the fitting process, ϵ_0 is the permittivity of vacuum in F/m, $m_n^* = 0.35 m_e$ is the effective electron mass in Kg, m_e is the electron mass in Kg, and e is the electron charge in Coulombs.

The measured carrier concentration and the estimated value obtained through ellipsometry fitting showed a good agreement per sample. For sample SR83, the ellipsometry-derived carrier concentration $|n_{b,ell}|$ is 3.37 $\times 10^{20}$, which differs by 1.5% from the carrier concentration measured using the Hall effect system. Similarly, for sample Hf3-227, the ellipsometry-derived carrier concentration $|n_{b,ell}|$ is 4.19 $\times 10^{20}$, exhibiting a difference of 5.5% compared to the carrier concentration measured using the Hall effect system.

The relative density of the hafnia layer can be estimated using the Lorentz-Lorenz (LL) equation. The relationship between the variation of the film density ρ_f and the refractive index of the fitted hafnia layer is described by the equation $\rho_f/\rho_{145} = (n_f^2 - 1)(n_{145}^2 + 2)/[(n_f^2 + 2)(n_{145}^2 - 1)]$, where ρ_{145} is the density and n_{145} is the refractive index of 145 nm thick hafnia reported in [3]. The LL equation is valid for a transparent film. Using $n_{145} = 1.878$ and $n_f = 1.851$ at $\lambda = 1.56$ μm, the relative density of the hafnia layer is $\rho_f/\rho_{145} = 0.97$ which indicates a good dense hafnia layer on the witness samples.

2 Illustrative Case of Phase Shift Measurement

Figure 1 displays the intensity of the interference pattern measured at 0 V and 4.5 V at 1560 nm wavelength, along with their corresponding Gaussian fittings. The intensity is presented in the digital scale of the IR camera. The peak values of the Gaussian fittings were utilized for calculating the phase shift. Initially, the value $A = \frac{\sqrt{I(0V)}}{2} = 287.5$ is computed. Subsequently, using the value of $I(4.5V) = 531$ in the phase shift equation $\phi(V) = \cos^{-1}\left(\frac{\sqrt{I(V)}-A}{A}\right)$:

$$\begin{aligned} \phi(4.5V) &= \cos^{-1}\left(\frac{\sqrt{I(4.5)} - A}{A}\right) \\ \phi(4.5V) &= \cos^{-1}\left(\frac{\sqrt{531} - 287.5}{287.5}\right) \\ \phi(4.5V) &= 22.8^{\circ} \end{aligned} \tag{4}$$

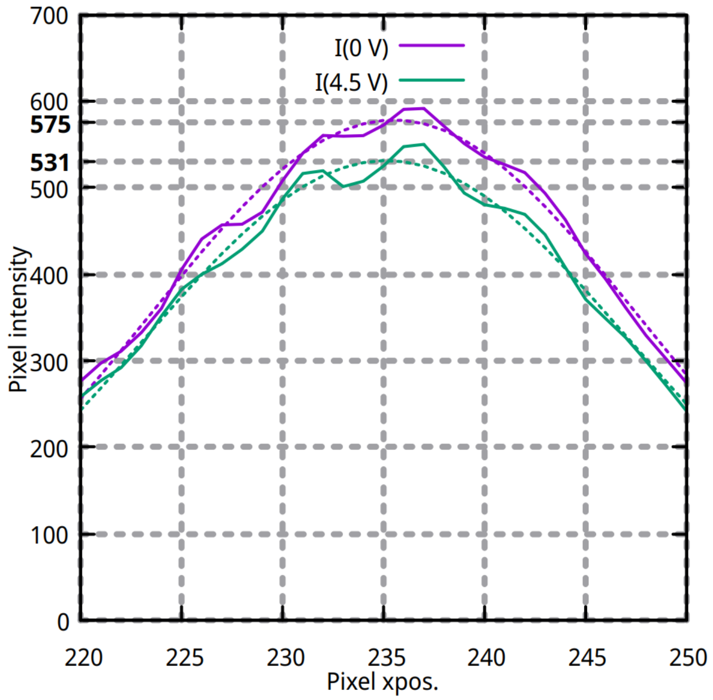

Fig. 1: Intensity of the interference pattern measured at 0 V and 4.5 V. The solid lines represent the raw measurement data, while the dashed lines corresponds to a Gaussian fittings.

3 Raw data measurements of the capacitance and consideration of the instrument accuracy

Table 1 displays the raw experimental measurements of capacitance per pin, including the corresponding offset from the zero value (ΔC). The offsets presented are in close proximity to the accuracy limit of our LCR meter, specified to be around 0.05 pF. Figure 2 illustrates the corresponding specific

capacitance calculated using the reported pin area, detailed further in this document, alongside the theoretical calculation for comparition.

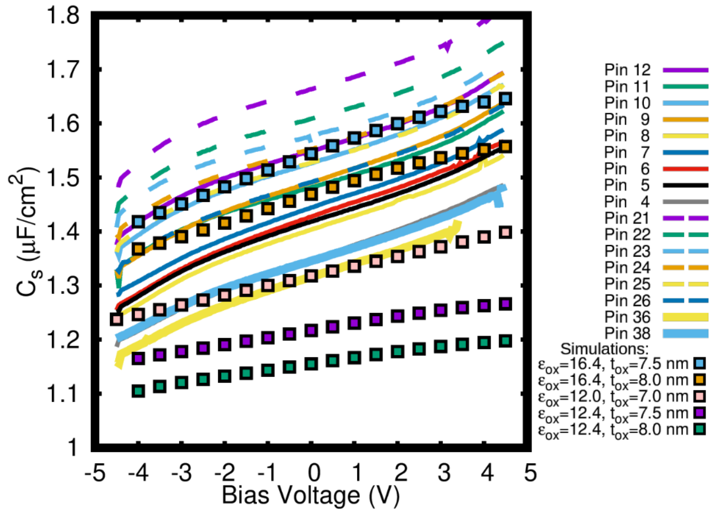

Fig. 2: Experimental capacitance per unir area of sub-wavelength reflectarray with parasitic capacitance offset of ≈ 0.1 $\mu F/cm^2$ along with calculated C-V characteristics.

Tab. 1: Raw capacitance measurements per pin.

Pin	C at 4.5V [pF]	ΔC (pF)
12	1.23692068047208	0.0730293896
11	1.16947351301844	0.0720869324
10	1.26052976060512	0.0754157948
9	1.26908116244908	0.0775309684
8	1.23367700351742	0.0800470418
7	1.30277889079296	0.0820291584
6	1.30803768792516	0.0834921226
5	1.31317071641776	0.0844330742
4	1.25982232351164	0.0847897002
21	1.32253303390016	0.0730293896
22	1.26111042148532	0.0720869324
23	1.29350908767116	0.0754157948
24	1.31274660385196	0.0775309684
25	1.33764210140726	0.0800470418
26	1.34026621618176	0.0820291584
38	1.11178718852108	0.0754157948

4 Pin serial resistance

Test structures were defined on the first metal layer to study the pin and fan-out resistance. Fig. 3(A) shows the test structure after deposition of the hafnia layer. Notably, fan-out fingers and pins 12, 21, 3 and 30 are short-circuited together. It is important to mention that fingers 3 and 30 have the same

dimensions (thickness, length, width), as do fingers 12 and 21, the former pair being of the shortest length and the latter pair being of the longest length.

The voltage-current characteristics of the pins were measured using a source-meter (2400, Keithley), which allows for voltage sourcing during measurements without the need for connection changes. A bias voltage was applied between pins 3 and 30 or between pins 12 and 21, and the resulting current was recorded. Fig. 3(B) displays the measured current for bias voltages ranging from -1 to 4 V. The I-V characteristics yield a consistent load 3.63 kΩ for the finger pair 3-30 and 3.68 kΩ for the finger pair 12-21. Based on these measurements, we determined that a single fan-out finger and pin resistance ranged from 1.81 to 1.84 kΩ.

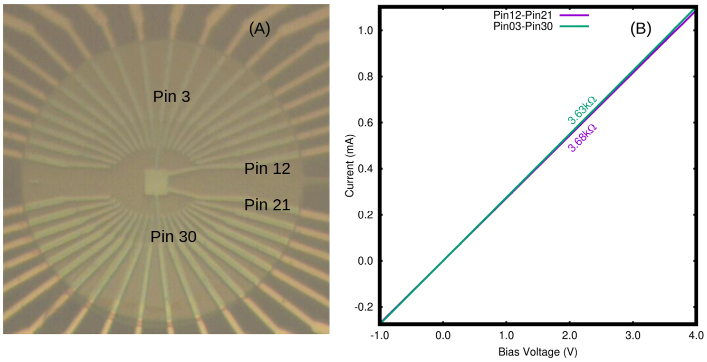

Fig. 3: (a)Test structure for DC testing. (b) Pin to pin current-voltage (I-V) characteristics.

5 Capacitor areas

The area of each capacitor was taken as the area of each fan-out finger beneath the ITO patch, as shown in Fig. 4. The areas were calculated from the layout file of the first metal layer. Moreover, the area of the nano-connectors from the fingers to the nano-array and the area of the nano-antennas were considered. Specifically, the area of the nano-connectors were 0.731524 μm^2. The area of the nano-antennas was calculated as $(10\ \mu\text{m}/a_y)[L_d W + (a_y - w)w_c] = 0.81327\ \mu\text{m}^2$, where the dimensions are in μm.

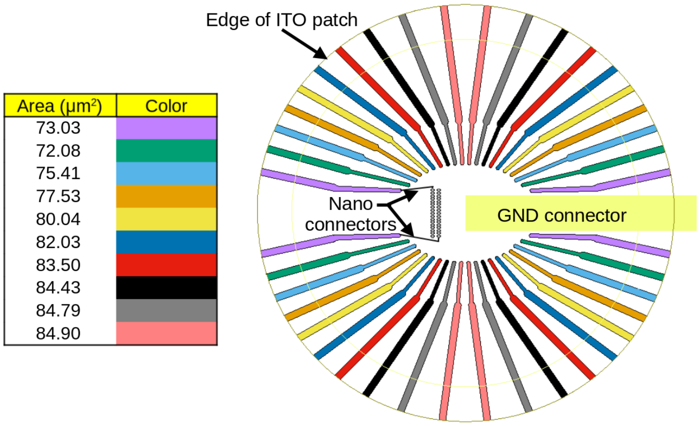

Fig. 4: Pin areas beneath the ITO patch.

References

[1] Patrick M. Hemenger, "Measurement of high resistivity semiconductors using the Van Der Pauw method", *Rev. Sci. Instrum.*, vol. 44, no. 698–700, 1973.
[2] C.Z. Tan, "Determination of refractive index of silica glass for infrared wavelengths by IR spectroscopy", *Journal of Non-Crystalline Solids*, vol. 223, no. 1–2, 1998.
[3] M.F. Al-Kuhaili, "Optical properties of hafnium oxide thin films and their application in energy-efficient windows", *Optical Materials*, vol. 27, no. 3, 2004.
[4] Zhaolin Lu, Wangshi Zhao and Kaifeng Shi, "Nanoscale plasmonic and optical modulators based on transparent conducting oxides", *arXiv*, vol. 1205, no. 0502, 2012.
[5] Justin W. Cleary, Evan M. Smith, Kevin D. Leedy, Gordon G Rzybowski And Junpeng Guo, "Optical and electrical properties of ultra-thin indium tin oxide nanofilms on silicon for infrared photonics", *Optical Materials Express*, vol. 8, no. 15, 2018.
[6] Masoud Shabaninezhad, Lora Ramunno and Pierre Berini, "Tunable plasmonics on epsilon-near-zero materials: the case for a quantum carrier model", *Optics Express*, vol. 30, no. 26, 2022.
